# Single-tuned passive filter (STPF) for mitigating harmonics in a 3-phase power system

**DOI:** 10.1038/s41598-023-47614-7

**Published:** 2023-11-25

**Authors:** Meshack Magaji Ishaya, Oluwatayomi Rereloluwa Adegboye, Ephraim Bonah Agyekum, Mohamed F. Elnaggar, Mohammed M. Alrashed, Salah Kamel

**Affiliations:** 1 Department of Electrical and Electronics Engineering, Cyprus International University, Mersin 10, North Cyprus Turkey; 2 Management Information System, University of Mediterranean Karpasia, Nicosia, Mersin-10, Turkey; 3 Department of Nuclear and Renewable Energy, Ural Federal University Named After the First President of Russia Boris Yeltsin, 19 Mira Street, Ekaterinburg, Russia 620002; 4 Department of Electrical Engineering, College of Engineering, Prince Sattam Bin Abdulaziz University, 11942 Al-Kharj, Saudi Arabia; 5 Department of Electrical Power and Machines Engineering, Faculty of Engineering, Helwan University, Hewlan, 11795 Egypt; 6 Department of Electrical Engineering, Faculty of Engineering, Aswan University, Aswan, 81542 Egypt

**Keywords:** Energy science and technology, Engineering

## Abstract

Numerous integrals of the fundamental frequency are known as harmonics and can be found in power systems or electrical circuitry systems. Non-linear loads occasionally drain current or contains a varying impedance with each period of the AC voltage are often responsible for power system harmonics. This can result in system overheating, system losses, and equipment or system damage. In order to achieve the IEEE 519 power quality standard, filters are routinely employed to lower harmonic levels. In this work, we designed a single tuned passive filter (STPF) to minimize harmonics of sequence 5th, 7th, 11th, 13th, 17th, and 19th in a three (3) phase power system. The measurements were taken at the point of common coupling. To test the filter performance, the system and STPF were designed in MATLAB/Simulink, and the simulated results produced without and with STPF were compared. The ${THD}_{I}$ was reduced from 14.93% down to 4.87% when STPF was connected which is within the IEEE 519-2022 standard; proving that the STPF was effective in decreasing the harmonics to the desired level.

## Introduction

**Figure 1:**
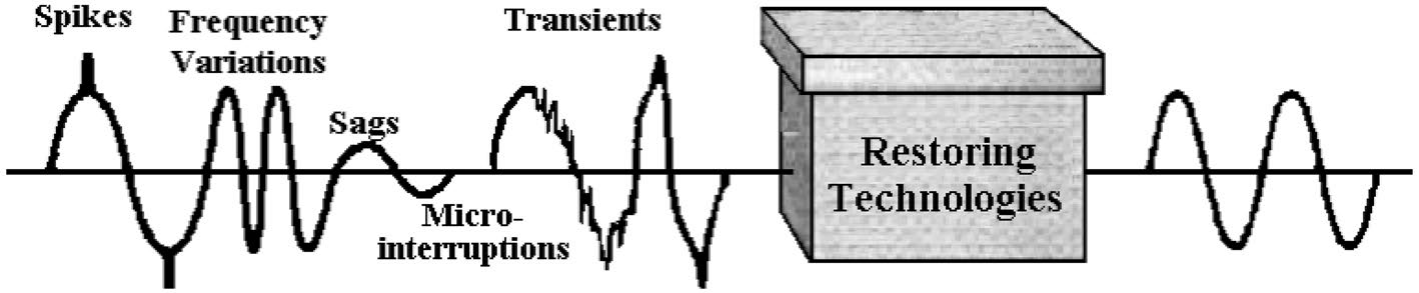
PQ issues and rectification^6^ (Published under open access).

When constructing both AC and DC distribution systems, power quality (PQ) is critical^1^. Control of power systems must be harmonious since PQ is a key study issue^2^. In IEEE 1100 standards, PQ is defined as the principle of powering and grounding sensitive electronic devices in a manner appropriate for the equipment^3^. PQ can also refer to the quality of power or voltage, which changes depending on the source and is merely the interface between the utility and the user. Although the voltage quality of a power system is commonly considered as the PQ, the voltage signal which is sinusoidal is reliant on the current signals, therefore analyzing the load’s current is critical. However, the electrical system's wires, cables, and other components experience voltage drops as a result of non-sinusoidal current flow, which distorts the sinusoidal voltage waveform^4^. Due to the widespread use of electronic devices, electric loads witnessed a tremendous change. These loads are the primary causes and victims of PQ problems, they disrupt the voltage waveform constituting a variety of disturbances^5^ due to their unpredictable nature^6^. When the loads change gradually, changes in the supply voltage can be easily controlled with voltage regulators. On the other side, when the loads impedance varies quickly, novel supply voltage occurrences, including sags, swells, notches, and flicker arise. The problem shifts from one of loading quality to one of supply quality^7^. Table 1 shows a few typical power quality issues and Fig. 1 shows some power quality issues and the need for a rectification technique.

**Table 1:** Common issues with power quality^6^ (Published under open access).

Categories	Explanation
Sag (dip)	AC voltage (RMS) drop in power system spanning a short duration usually often under a second
Swell	RMS increase in AC voltage at power system within a few seconds to a cycle
Transients	Resulting from abrupt current or voltage fluctuations within a power system. The power transmission resistance, inductance, and capacitance the region of emphasis commonly affect the characteristics of transients, which are brief-lived phenomena
Harmonics	The sinusoidal component of a wave that is periodic having a frequency that is directly proportional to the primary frequency
Distortion	Denotes the variation of periodic wave from its standard waveform features
Impulse	In the past it was referred to as transient overvoltage event with particular peak and fall characteristics. It is becoming more widely accepted that the word "impulse" refers to transients
Noise	unacceptable electrical impulses that, when used in control system circuits, produce unpleasant results
Notch	Natural power voltage waveform disruption that commences with the waveform's opposite polarity and decreases from it as a consequence, lasting a fraction of a cycle
Power disturbance	Any deviation from the standard quantity of input AC features
Coupling	Energy or electrical interference transfer from a particular circuit to a subsequent one physically connected
Flicker	a shift in the voltage of the input lasting sufficiently much for the eye to observe a change an electric light source's brightness
Form factor	Ratio of the average to RMS values of a periodic waveform. Another indicator of the extent to which a periodic waveform deviates from its most desirable features

Numerous efforts have recently been carried out to improve the quality of components affected by harmonics in a variety of ways, including functionality and efficiency enhancement, harmonic suppression, and downsizing^8^.

This paper begins with an introductory section that outlines several PQ concerns. In “Harmonics” section, harmonics is the most serious PQ issue; passive filters as a mitigating model; related work on the use of passive filters for harmonics mitigation and their effectiveness in comparison with the IEEE 519 standard were discussed. STPF; its configuration, function, parameters, and mathematical model was captured in “Methodology” Section. In “Methodology” section also describes the approach, which includes a flowchart, measurements, and a mathematical model for computing various STPF parameters, as well as the MATLAB/Simulink simulation platform. The findings of the study were addressed in “Result and discussion” section, and lastly, a conclusion and recommendation are presented in “Conclusion” section.

## Harmonics

**Figure 2:**
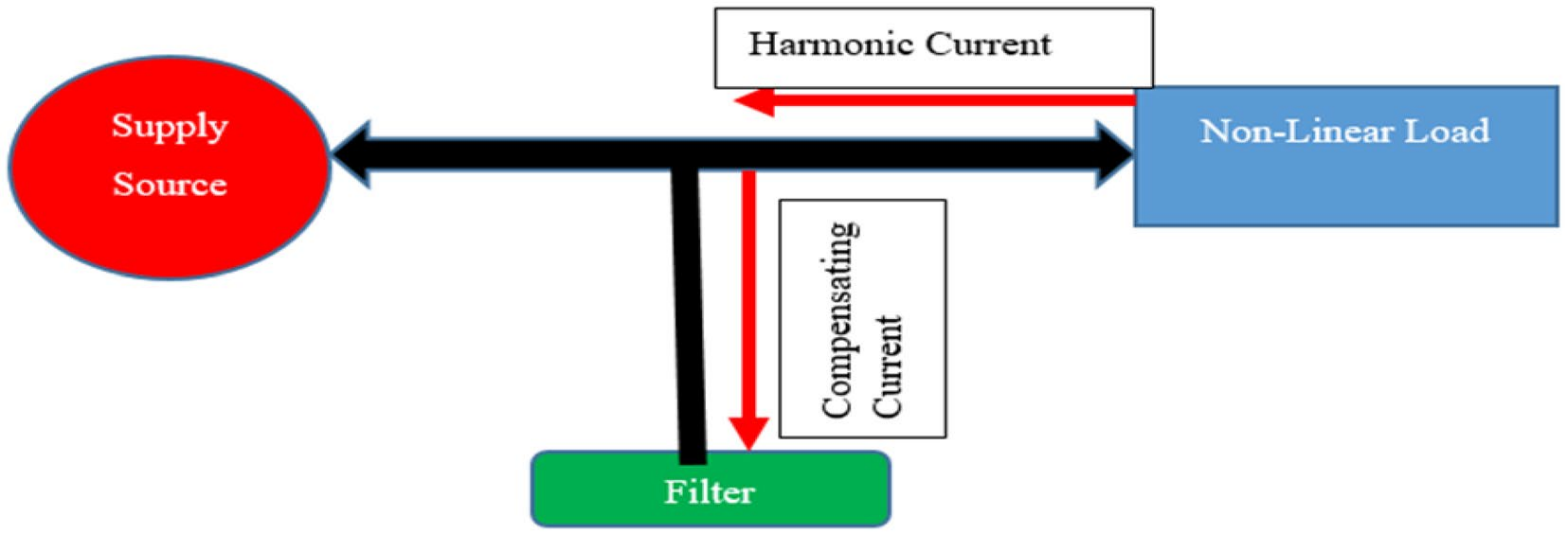
Harmonic current flowing because of a non-linear load.

Among the listed power quality issues, harmonics is the most concern. It is considered be the multiple integral of fundamental frequency^9^. As a result of a rise in non-linear loads, harmonic issues have recently become more important^10,11^. As a consequence of advancements in technology, including the usage of power electronic circuits, equipment for AC to DC transmission connections, loads for power electronics-based power system regulation, and microprocessor controllers^12^, and renewable energy applications^13^. A substantial quantity of harmonic distortion in the power systems is caused by the expanding use of distributed, renewable, intermittent power sources, the spread of power electronic equipment and nonlinear loads which have high levels of uncertainty^14^. The two main effects of PQ problems on the transmission network appears to be the possibility for equipment failure to negatively impact efficiency and the drop in power factor caused by specific forms of harmonics. Figure 2, demonstrates harmonic current flow through all the system's impedances towards the source from a non-linear load with a filter connected to turn the harmonic current away from the sources and toward ground.

A key PQ issue in power systems, harmonic distortion, might lead to more breakdowns of equipment and communications interference^15^, fuse and breaker malfunction, transformer overheating, device malfunction^16^, conductor heating, power loss, decrease in power factor and financial losses^17^. To completely control harmonic interference, since it cannot be completely removed from the system because some of the harmonics are created by system equipment. Users' ability to generate a certain quantity of harmonic current is constrained by the IEEE 519 standard. Systems of rewards and penalties are applied to consumers according to the amount of harmonic current they emit. The technique is put into practice after each harmonic source has been fairly and openly evaluated. Table 2 displays a few system consequences of current and voltage distortion, while the main effects of harmonic current and voltage on the power system are shown in Table 3.

**Table 2:** Some system distortion caused by voltage and current.

Total harmonic distortion of voltage (${{\varvec{T}}{\varvec{H}}{\varvec{D}}}_{{\varvec{V}}})$	Total harmonic distortion of current (${{\varvec{T}}{\varvec{H}}{\varvec{D}}}_{{\varvec{I}}}$)
${{\varvec{T}}{\varvec{H}}{\varvec{D}}}_{{\varvec{V}}}=\frac{1}{{{\varvec{V}}}_{1}}\surd {{{\varvec{\Sigma}}}_{{\varvec{n}}=2}^{\boldsymbol{\infty }}{{\varvec{V}}}_{{\varvec{n}}}}^{2}$	${THD}_{I}=\frac{1}{{I}_{1}}\surd {{\Sigma }_{n=2}^{\infty }{I}_{n}}^{2}$
Insulation stress	Increase power loss
Load disruption	Transformer secondary voltage distortion
Thermal stress	Telephone and communication system noise
	Overload neutrals and capacitors
	Transformer heating

**Table 3:** Impacts pf current and voltage harmonics in power network.

(1) Series and parallel resonances result in an increase in harmonic rates
(2) Electrical energy is produced, transmitted, and used in less effective ways
(3) The decline in the insulation of electrical system elements, which shortens the time they last
(4) System or industrial component failure

### Passive filter

Passive filter can mitigate most of the PQ issues^18^. It has an advantage of low cost, simplicity, flexibility^19^ and comes in variety of configurations^20^ with various frequency response properties^21^. One of the efficient methods used to stop or slow the spread of harmonics in the electrical grid is the use of passive harmonic filters. Compared to active filters, passive filters are more stable, capable of withstanding huge currents, and substantially less expensive^22^. The STPFs, double tuned passive filters, damped filters (high and band-pass, second-order, third-order), C-type passive filter, and composite passive filter^11,23,24^ are all types of passive filters. In literature, several studies on filter setup in dispersed networks have been conducted^25^.

In the study of Mambidala et al.^10^, a new three-phase single tuned harmonic filter was developed to improve the utility grid's voltage profile, current profile, and total harmonic distortion. ${THD}_{I}$ was measured from the current source obtaining 16.5% without filter and after the proposed new three-phase tuned harmonic filter was introduced the ${THD}_{I}$ reduced to 0.02% establishing the effectiveness of the filter in mitigating harmonics. Furthermore, it was observed that when three, two and one electric vehicle(s) (EV) batteries were connected, the ${THD}_{V}$ without filter was 11.82%, 20.60% and 28.87%, respectively, and after the filter was connected the ${THD}_{V}$ dropped to 0.18%, 0.0%, and 0.0% when three, two and one EV batteries were connected, respectively.

Melo et al.^15^, outlined an optimization issue for sizing and placing STPFs in power supply networks with the aim of reducing overall distortion from harmonics. The ${THD}_{V}$ before the utilization of filter were as follows: bus 63,${THD}^{a}\left(\%\right)=6.060$, ${THD}^{b}\left(\%\right)=5.3933$, ${THD}^{c}\left(\%\right)=4.8699$, after the filter was applied, 63, ${THD}^{a}\left(\%\right)=3.2062$, ${THD}^{b}\left(\%\right)=4.1038$, ${THD}^{c}\left(\%\right)=2.9476$. This clearly shows that the single tuned passive filter was effective.

Similarly, in the study of Anuar and Abdullah^16^, at the point of common coupling (PCC), a passive power harmonic filter was used to remove notable current harmonics (5th, 7th, and 11th) from a 240 V (rms) utility. The ${THD}_{V}$ values for phase A, B, and C systems without filters are 24.36%, 29.19%, and 29.39%, respectively. When the filter was connected, ${THD}_{V}$ dropped to 7.83% in phase A, 10.78% in phase B, and 10.24% in phase C.

The study of^18^ presented a single phase shunt active filter supported by controllers. At steady state, the grid’s current ${THD}_{I}$ with the three controllers proportional integral to low pass filter (PI-LPF), PI-dual self-tuning filter (PI-DSTF) and back stepping controller-DSTF (BSC-DSTF) are observed at 7.5%, 4.2% and 4.2%, respectively. The grid current ${THD}_{I}$ is improved with the DSTF controller within the IEEE 519 standard.

Also, Bajaj and Singh^23^ employed the firefly algorithm (FA) to address the design of various passive power filter (PPF) types, which is formulated as an optimization problem with the goal of increasing the penetration of a renewable distribution system (DG) in a distorted distribution power grid. The filters were ranked from less effective to most effective as composites types in terms of their performance and the percentage of penetration level after simulation was carried out; single-tuned, second-order damped, C-type, and third-order damped. In time of high cost, the order reverses.

In the work of Khattab et al.^11^, a mathematical model of a novel fourth-order harmonic passive filter damped high pass filter (DHPF) is proposed alongside considering various design scenarios. The crow spiral based search algorithm (CSSA) is applied to solve the design issues. ${THD}_{I}$ and ${THD}_{V}$ without filter are 5.13% and 6.55% respectively. The second order filter was applied the ${THD}_{I}$ and ${THD}_{V}$ reduced to 4.36 and 3.16, respectively, third order filter produced ${THD}_{I}$ and ${THD}_{V}$ of 4.18 and 3.15, respectively. The C-type filter generated ${THD}_{I}$ and ${THD}_{V}$ of 4.47 and 3.10, respectively. The proposed DHPF obtained ${THD}_{I}$ and ${THD}_{V}$ value of 4.18% and 2.90, respectively, demonstrating the effective of the filter within the IEEE-519 standard.

Harmonic is a significant challenge to power systems that cannot be completely eliminated but can be mitigated. The reason for this is that some of the power equipment used for power system setup cause some losses themselves, and because the equipment comprises a significant portion of the system and is required for the power system to operate, the equipment cannot simply be removed from the system. The solution is to reduce the rate of losses caused by the equipment in the power system.

In this paper, we proposed STPF as a harmonic mitigation technique is employed after identifying harmonics as a key issue impacting PQ because of its advantages of low component cost, compact size, simplicity in design, affordable, and improves power quality. The novelty of our paper, which is presented in the design of STPF for a specific three phase plastic processing industry power system to mitigate harmonics of the following sequence 5th, 7th, 11th, 13th, 17th, and 19th adaptable for high frequency applications that is tested for effective harmonics mitigation in accordance with the IEEE 519-2022 standard (advanced of both IEEE 519-1992 and IEEE 519-2014)^26^.

## Methodology

### STPF

**Figure 3:**
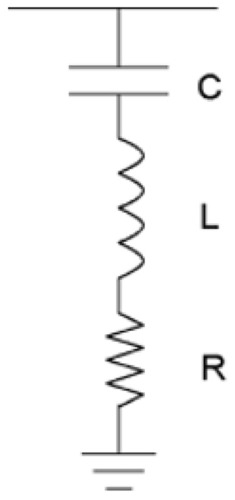
STPF configuration.

A STPF is meant to filter out a single harmonic, and is straightforward to develop^13^ inexpensively with components that are all connected in series: a resistor (R), an inductor (L), and a capacitor (C)^27^, which provides low impedance path^28^. It enables harmonic current to flow across and reach the ground as shown in Fig. 2. In this study, STPF was used as a corrective measure to reduce the harmonics in the power system. Figure 3 shows the configuration of STPF.

In STPF design, the following essential parameter were considered R, C, L, $X_{c}$ and $Q_{c}$. Where $X_{c}$ denotes capacitance reactance,$Q_{c}$ reactive power to improve power factor (Q) and prevent the electric power provider from imposing any fines. In most cases, Q adjustment is performed to raise the Q to around 0.98 or above.

1 $$X_{C} = \frac{{V_{C}^{2} }}{{Q_{C} }}$$

2 $$C = \frac{1}{{2\pi f_{0} X_{c} }}$$

3 $$Q = \frac{{X_{n} }}{R} = \frac{{\sqrt {\frac{{L_{STPF} }}{{C_{STPF} }}} }}{R},\;{\text{assuming the power quality factor }}(Q) = 100$$

4 $$Q_{c} = P\{ \left[ {\tan (\cos^{ - 1} p_{f1} } \right)] - \left[ {\tan (\cos^{ - 1} p_{f1} } \right)]\} KVAR$$

5 $$p_{f} = \cos \theta$$

6 $$\theta = \cos^{ - 1} p_{f}$$

7 $$Q_{c} = P\left( {\tan \theta_{1} - \tan \theta_{2 } } \right)$$

where power factor $(p_{f} )$, capacitance reactive power $(Q_{c}$), capacitance $\left( C \right)$, active power $\left( P \right)$

8 $$X_{L} = 2\pi fL,\;{\text{inductance reactance}}$$

9 $$L = \frac{{X_{L} }}{2\pi f} = 9.86182\;{\text{mH}},\;{\text{inductor}}$$

10 $$X_{n} = \sqrt {X_{L} X_{C} } = \sqrt{\frac{L}{C}} = 3.09811,\;{\text{characteristic reactance}}$$

11 $$R = \frac{{X_{n} }}{Q} = 0.03097\,\Omega ,$$

12 $$Z = Z_{R} + Z_{L} + Z_{C} = R + j\left( {X_{L} - X_{C} } \right),\;{\text{impedance}}$$

STPF has a predisposition to engage with the system, testing every imaginable interaction with the system is especially useful. For safety reasons, STPF is tuned slightly below the harmonic frequency. In the presence of mutual inductances, the tuning of the STPF to eliminate specific harmonics is not done adequately, and the filter's efficiency is diminished in practice^29^.

**Figure 4:**
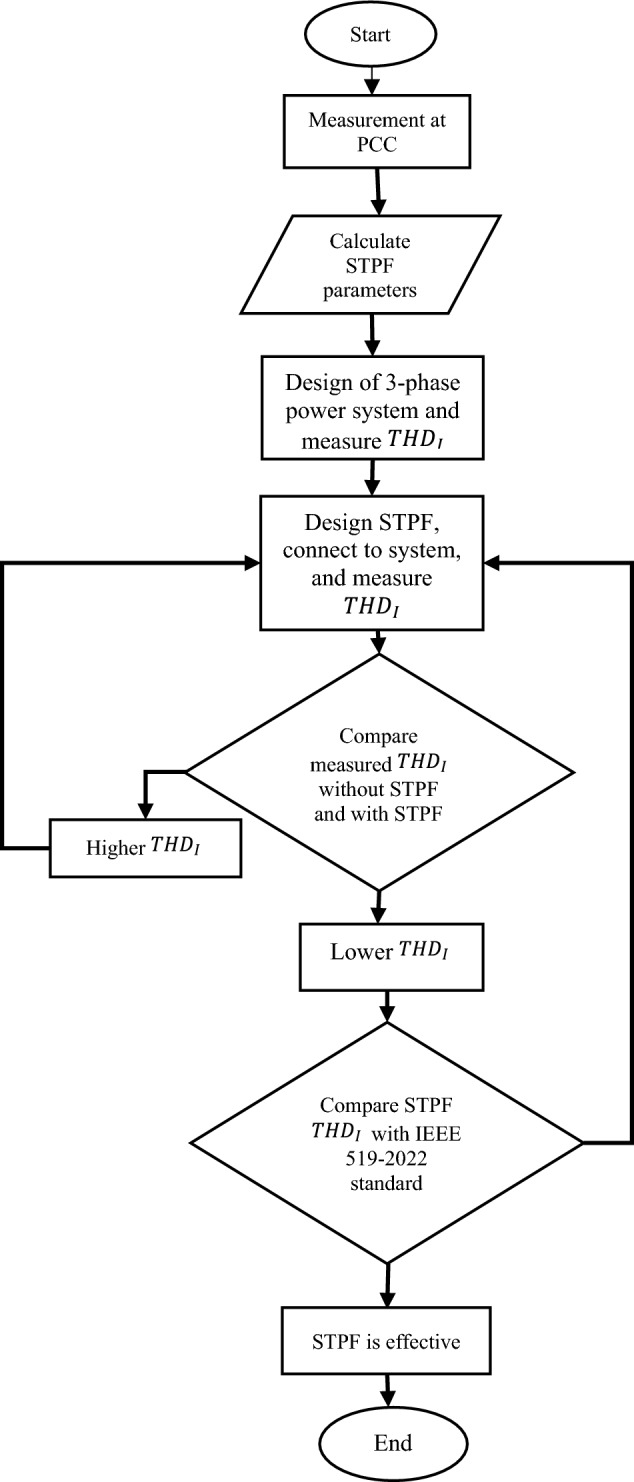
A flow chart demonstrating the methodology flow.

The following are the processes required to implement the proposed method as demonstrated in the flow chart in Fig. 4:

i. Measurement from PCC is obtained.
ii. STPF parameters R, L, and C are calculated.
iii. The power system is designed using MATLAB/Simulink.
iv. Measure ${THD}_{I}$ without STPF in the system.
v. Design STPF, connect to the 3-phase power system and measure ${THD}_{I}$.
vi. Compare ${THD}_{I}$ without and with STPF.
vii. Compare ${THD}_{I}$ with STPF and IEEE 519-2022
viii. STPF is effective if ${THD}_{I}$ with STPF is equal to or less than IEEE 519-2022 standard.
ix. The effectiveness of STPF is determined.

### Measurements and calculations

Metrel MI 2392 Power Q Plus Power Quality Analyzer was used to measure current, voltage, active power, reactive power, apparent power and harmonics at PCC^30^, the following results were obtained as shown in Table 4 from a balanced three phase load and analysis was carried out considering one phase. The ${THD}_{I}$ content in plastic processing industry load is 14.93%, as shown in Table 4 which exceeds the IEEE 519-2022 limit of less than 5%. This paper proposes a STPF that will reduce the ${THD}_{I}$ to the IEEE 519-2022 benchmark. The estimations for the RLC component are presented in Table 5.

**Table 4:** Data acquired using the power quality analyzer tool on the PCC pane^31^ (Published under open access).

Names	Phase values		
	Symbols	Values	Unit
Voltage	V	234.45	V
Current	I	269.94	A
Voltage total harmonic distortion	${THD}_{V}$	5.1187	%
Current total harmonic distortion	${THD}_{I}$	14.93	%
Active power	P	80.508	kW
Reactive power	Q	29.65	kVAR
Apparent power	S	85.794	kVA
Power factor	${P}_{f}$	0.94

**Table 5:** Calculation results for the RLC Component.

Resistance (Ω)	Capacitance (µF)	Inductance (MH)
0.03097	1028.45	9.86182

Supposedly, ${P}_{f}$ changes from ${p}_{f1}$ = 0.94 to ${p}_{f2}$ = 0.99. These numbers are used to calculate the capacitor capacity (${Q}_{c}$).

$$\theta_{1} = 19.95$$, from (6).

$$\theta_{2} = 8.11$$, from (6).

$$Q_{c}$$ = 17.75066 $$KVAR$$ using (9).

$$X_{C} = \frac{{V_{C}^{2} }}{{Q_{C} }}$$ = $$\frac{{234.45^{2}_{c} }}{17750.66}$$ = 3.09661 Ω, using (1).

$$C$$ = 1028.45 µF, applying (2).

$$X_{L} = X_{C}$$ = 3.09661 Ω at resonance.

## Result and discussion

**Figure 5:**
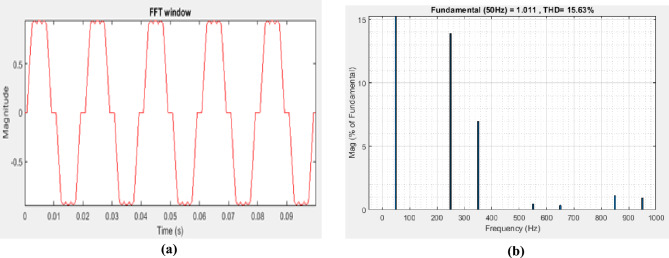
(**a**) Current signal aberration, (**b**) ${THD}_{I}$ preceding system's application of the filter.

Figure 5a without connecting STPF shows the distorted current waveform with ripple at it top nearly square appearance not quite sinusoidal, due to harmonics was obtained by MATLAB/Simulink simulation model. Figure 5b demonstrates the sequence of the harmonics having a primary frequency of 50 Hz and ${THD}_{I}$ value of 15.63% which is above the IEEE 519-2022 rate, showing the requirement for a filter to minimize the ${THD}_{I}$ to the accepted level. Harmonics of sequence 5th, 7th, 11th, 13th, 17th, and 19th stood out amongst others as shown in Fig. 5b.

**Figure 6:**
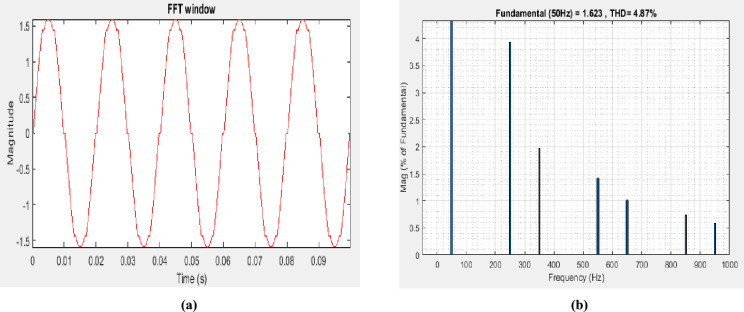
(**a**) Waveform of the current after applying STPF, (**b**) ${THD}_{I}$ after STPF was used to reduce 5th, 7th, 11th, 13th, 17th, and 19th sequence.

The simulation outcome when STPF was employed demonstrate the improvement of the magnitude of the current waveform as shown in Fig. 6a and the decrease in ${THD}_{I}$ from 15.63 to 4.87% as illustrated in Fig. 6b, accordingly. The ${THD}_{I}$ outcome from the simulation conforms to the IEEE 519-2022 standard.

**Figure 7:**
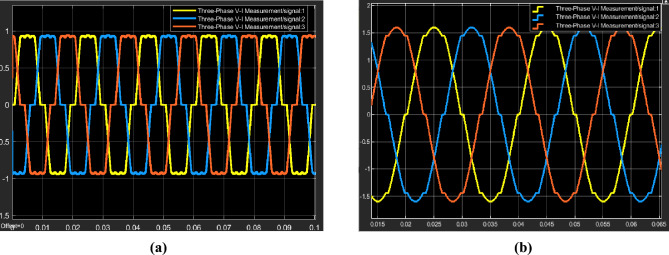
(**a**) Current distortion due to non-linear load, (**b**) STPF filter effect of current signal.

Figure 7a shows the deformed current waveform with ripples at its top, while Fig. 7b shows the nearly perfect sinusoidal waveform that resulted from the STPF's influence.

Table 6 compares the distinct harmonics of particular order considering measurements before and after connecting the STPF as well as STPF and the IEEE 519-2022 standard. Table 6's outcome in column four demonstrates that both the individual and $({THD}_{I}$) were in compliance with the standards following the application of STPF.

**Table 6:** Distinct and ${THD}_{I}$.

Harmonic order	Measurement from PCC in %^31^	Simulation result without STPF in %	Simulation result with STPF in %	IEEE 519-2022 standard in %
1st	100	100	100	
5th	14.22	13.89	3.94	4.0
7th	2.35	6.95	1.97	4.0
11th	1.21	0.49	1.44	2.0
13th	1.03	0.35	1.01	2.0
17th	0.84	1.12	0.73	1.5
19th	1.02	0.90	0.59	1.5
Total (${THD}_{I}$)	14.93	15.63	4.87	5.0

**Figure 8:**
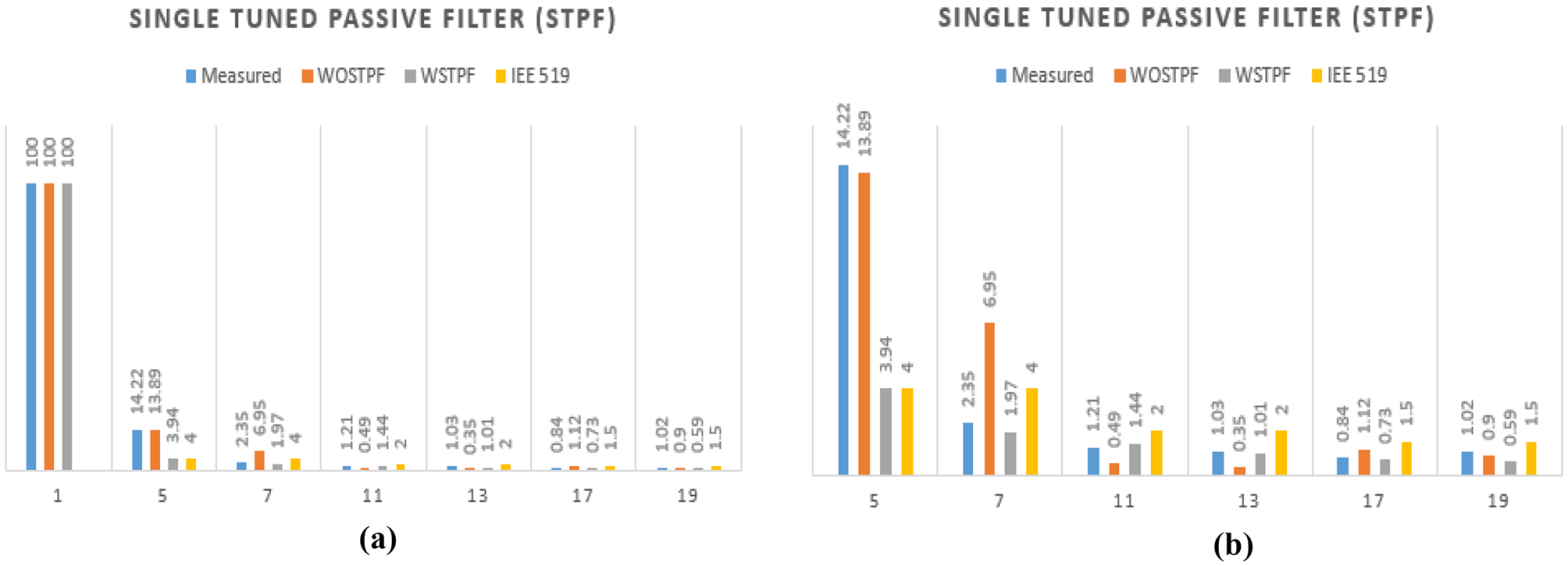
A bar chart of the results obtained at PCC for WOSTPF, WSTPF, and IEEE 519-2022 sequences (**a**) 1st, 5th, 7th, 11th, 13th, 17th, and 19th, (**b**) 5th, 7th, 11th, 13th, 17th, and 19th.

Figure 8a and b provide a plot of the results of Table 6, highlighting the diversity in each of the unique harmonic orders. Figure 8a shows the entire harmonics including the fundamental harmonic while in Fig. 8b, sequence 5th, 7th, 9th, 11th, 13th, and 19th without the 1st order harmonic were captured. The bar in blue color denotes the measured value at the PCC, the red colored bar denotes the individual harmonic without STPF (WOSTPF) connected, the ash-colored bar denotes the individual harmonic with STPF (WSTPF) connected, and the yellow-colored bar represents the IEEE 519-2022 standard.

## Conclusion

The power system’s ${THD}_{I}$ value obtained was 15.63% without STPF connected to the 3-phase power system, with the connection of STPF the value was reduced from 15.63 to 4.87% within the IEEE 519-2022 standard which shows the effectiveness of the STPF filter used in minimizing harmonics in the power system. The STPF was successfully tuned to mitigate harmonics of sequence 5th, 7th, 11th, 13th, 17th, and 19th. Difficulties encountered when designing the system are particularly connected to the individual and overall harmonic distortion limits for both current and voltage at the PCC. STPF was only able to eliminate harmonics one at a time and was developed with a fixed nonlinear load, despite the fact that the load frequency can rapidly change.

### Recommendation

STPF is unable to deal with frequency variations in the load, fails to eliminate frequencies other than the adjusted ones, and has issues with series/parallel resonance resulting to the need of more filters. To produce a superior overall result, a double tuned passive filter, damped filters or hybrid harmonic filter combination of an active–passive filter may be required. The optimization technique can be used to find the optimal STPF sizing by taking into consideration the total filter size due to changes in THD and determining the best R, L, and C for the filter.

## Data Availability

All data generated or analyzed during this study are included in this published article.
